# A novel imaging marker of cortical “cellularity” in multiple sclerosis patients

**DOI:** 10.1038/s41598-024-60497-6

**Published:** 2024-04-29

**Authors:** Muhamed Barakovic, Matthias Weigel, Alessandro Cagol, Sabine Schaedelin, Riccardo Galbusera, Po-Jui Lu, Xinjie Chen, Lester Melie-Garcia, Mario Ocampo-Pineda, Erik Bahn, Christine Stadelmann, Marco Palombo, Ludwig Kappos, Jens Kuhle, Stefano Magon, Cristina Granziera

**Affiliations:** 1 Translational Imaging in Neurology (ThINK) Basel, Department of Biomedical Engineering, Faculty of Medicine, University Hospital Basel and University of Basel, Basel, Switzerland; 2 Department of Neurology, University Hospital Basel, Petersgraben 4, 4031 Basel, Switzerland; 3 Research Center for Clinical Neuroimmunology and Neuroscience Basel (RC2NB), University Hospital Basel and University of Basel, Basel, Switzerland; 4 Pharmaceutical Research and Early Development, Roche Innovation Center Basel, F. Hoffmann-La Roche Ltd., Basel, Switzerland; 5 Institute of Neuropathology, University Medical Center, Göttingen, Germany; 6 School of Psychology, Cardiff University Brain Research Imaging Centre (CUBRIC), Cardiff University, Cardiff, UK; 7 School of Computer Science and Informatics, Cardiff University, Cardiff, UK

**Keywords:** Multiple sclerosis, Translational research, Biophysical models

## Abstract

Pathological data showed focal inflammation and regions of diffuse neuronal loss in the cortex of people with multiple sclerosis (MS). In this work, we applied a novel model (“soma and neurite density imaging (SANDI)”) to multishell diffusion-weighted MRI data acquired in healthy subjects and people with multiple sclerosis (pwMS), in order to investigate inflammation and degeneration-related changes in the cortical tissue of pwMS. We aimed to (i) establish whether SANDI is applicable in vivo clinical data; (ii) investigate inflammatory and degenerative changes using SANDI soma fraction (*f*_*soma*_)—a marker of cellularity—in both cortical lesions and in the normal-appearing-cortex and (iii) correlate SANDI *f*_*soma*_ with clinical and biological measures in pwMS. We applied a simplified version of SANDI to a clinical scanners. We then provided evidence that pwMS exhibited an overall decrease in cortical SANDI *f*_*soma*_ compared to healthy subjects, suggesting global degenerative processes compatible with neuronal loss. On the other hand, we have found that progressive pwMS showed a higher SANDI *f*_*soma*_ in the outer part of the cortex compared to relapsing–remitting pwMS, possibly supporting current pathological knowledge of increased innate inflammatory cells in these regions. A similar finding was obtained in subpial lesions in relapsing–remitting patients, reflecting existing pathological data in these lesion types. A significant correlation was found between SANDI *f*_*soma*_ and serum neurofilament light chain—a biomarker of inflammatory axonal damage—suggesting a relationship between SANDI soma fraction and inflammatory processes in pwMS again. Overall, our data show that SANDI *f*_*soma*_ is a promising biomarker to monitor changes in cellularity compatible with neurodegeneration and neuroinflammation in the cortex of MS patients.

## Introduction

Cortical pathology in multiple sclerosis (MS) is present at all disease stages and is extensive in progressive MS forms^1–5^. The presence of focal cortical damage in MS patients is clinically relevant because it is predictive of clinical disease progression (for both physical disability and cognitive decline^6–9^) and of conversion to secondary progressive MS^8–14^. Therefore, a deep understanding and characterization of cortical damage in MS patients are fundamental for identifying and preventing the insidious manifestations of clinical worsening in people with MS (pwMS).

Focal cortical lesions in pwMS have been categorized based on their location: some involve both the cortical grey and the underlying white matter (leukocortical lesions, type I)^15^; others are found deep within the cortical ribbon (intracortical lesions, type II); the most frequent ones involve the cortex starting from its pial surface and extending below, sometime till covering the entire cortical thickness (subpial lesions, type III and IV)^16,17^.

Histopathologically, cortical lesions (CLs) exhibit less inflammatory infiltrates in comparison to white matter lesions (WMLs) but may show variable amounts of immune cells, such as myelin-laden macrophages, non-active and active microglia; CLs also contain damaged and/or apoptotic neurons and remyelinating oligodendrocytes^18–22^. Especially subpial lesions have been shown to harbor activated microglia cells that are thought to trigger and perpetuate demyelination^10,23^. In fact, B cells and other immune cells in the meninges may produce factors that diffuse into the cortical parenchyma and activate microglia, a phenomenon associated with ongoing demyelination and axonal injury in cortical plaques^3,18,24^ as well as in the normal-appearing cortex^10,24^. Neuronal loss in the cortex of MS patients might result from intracortical inflammation—especially in subpial lesions—as well as the retrograde degeneration of axons injured in WMLs—for example, in leukocortical lesions^25^ and in non-lesion areas with extended axonal projections^26–28^.

The characteristics of cortical lesions are currently unexplored with MRI due to the lack of contrast sensitivity and specificity. Positron emission tomography (PET) allows to identify microglia activation in CLs^29^. However, PET studies are associated with ionizing radiation exposure^30^, limited spatial resolution^31^, and high-infrastructural needs^32^, limiting their applicability to large-scale investigations of cortical pathology in MS.

Some recent mathematical models applied to Diffusion-Weighted Magnetic Resonance Imaging (DW-MRI)^33^ data have shown promise in quantifying the cellular and axonal compartments of the brain tissue, opening new perspectives to investigate the inflammatory characteristics and neurodegenerative consequences of inflammation with an unprecedented level of precision.

Neurite Orientation Dispersion and Density Imaging (NODDI)^34^ and 3D Anisotropic MicrOstructural eNvironments in Diffusion-compartment imaging (DIAMOND)^35^, for example, have provided measures related (i) to white matter (WM) microglia density in mice treated with colony-stimulating factor 1 receptor (CSF1R) inhibitor^36^ as well as (ii) to microglia activation in rats that underwent dorsal root axotomy^37^. Another multicompartment model that was recently applied to advanced DW-MRI data obtained in a human 3T CONNECTOM scanner (to date, the most powerful research-only DW-MRI 3T scanner equipped with 300 mT/m diffusion gradient strength), have supported with measures that are related to the presence of activated microglia and astrocytes in healthy human brains^38^.

Different from the models above, the single diffusion time *apparent cell body (namely SomA) and Neurite Density Imaging* (SANDI)^39^ model has been specifically adapted to the cortical grey matter tissue, thereby supporting with a measure related to tissue cellularity, i.e., SANDI soma fraction (*f*_*soma*_). The parameter *f*_*soma*_ represents the fraction of the signal that is attributed to the soma compartment in the SANDI model. This model was designed to capture the microstructural complexity of brain tissue by considering different compartments, including soma (cell bodies), neurites (axons and dendrites), and the extracellular space. This parameter is particularly relevant when assessing cortical regions where the density of cell bodies is high. SANDI *f*_*soma*_ has been shown to correlate with neuronal soma density in ex-vivo^39^ and in-vivo^40^ mouse data. In humans, it was tested on data acquired on a non-clinical 3T CONNECTOM scanner^39^. Furthermore, was acquired for the first time with a clinical scanner (3T Philips Ingenia CX) with a customized DW-MRI sequence^41^ that mimics the original protocol.

In this study, we aimed first to assess the feasibility of applying SANDI to data acquired in a clinical Siemens Magnetom Prisma 3T MRI system, relaxing both the model and the DW-MRI sequence required; then, we investigated the relationship between SANDI measures and other quantitative MRI measures obtained at 3T, such as Magnetization Transfer saturation and T1 relaxometry. Subsequently, we explored the presence of inflammatory and degeneration-related phenomena in the cortex of MS patients using cellularity-related metrics provided by SANDI (i.e., SANDI *f*_*soma*_). Last, we investigated the relationship between SANDI *f*_*soma*_ and biological and clinical measures of disease impact.

## Results

### Numerical simulations of SANDI resolution in CONNECTOM and Prisma MRI scanner

The SANDI^39^ model was developed and validated on preclinical, experimental mouse brain data obtained at 9.4 Tesla^40^ and in vivo in humans using a research 3T MRI CONNECTOM scanner^42^, which is equipped with a maximum diffusion gradient strength of 300 mT/m.

Our study aimed to apply SANDI to the data of a clinical Siemens Magnetom Prisma 3T MRI scanner, which has a maximum diffusion gradient strength of 80 mT/m.

**Figure 1:**
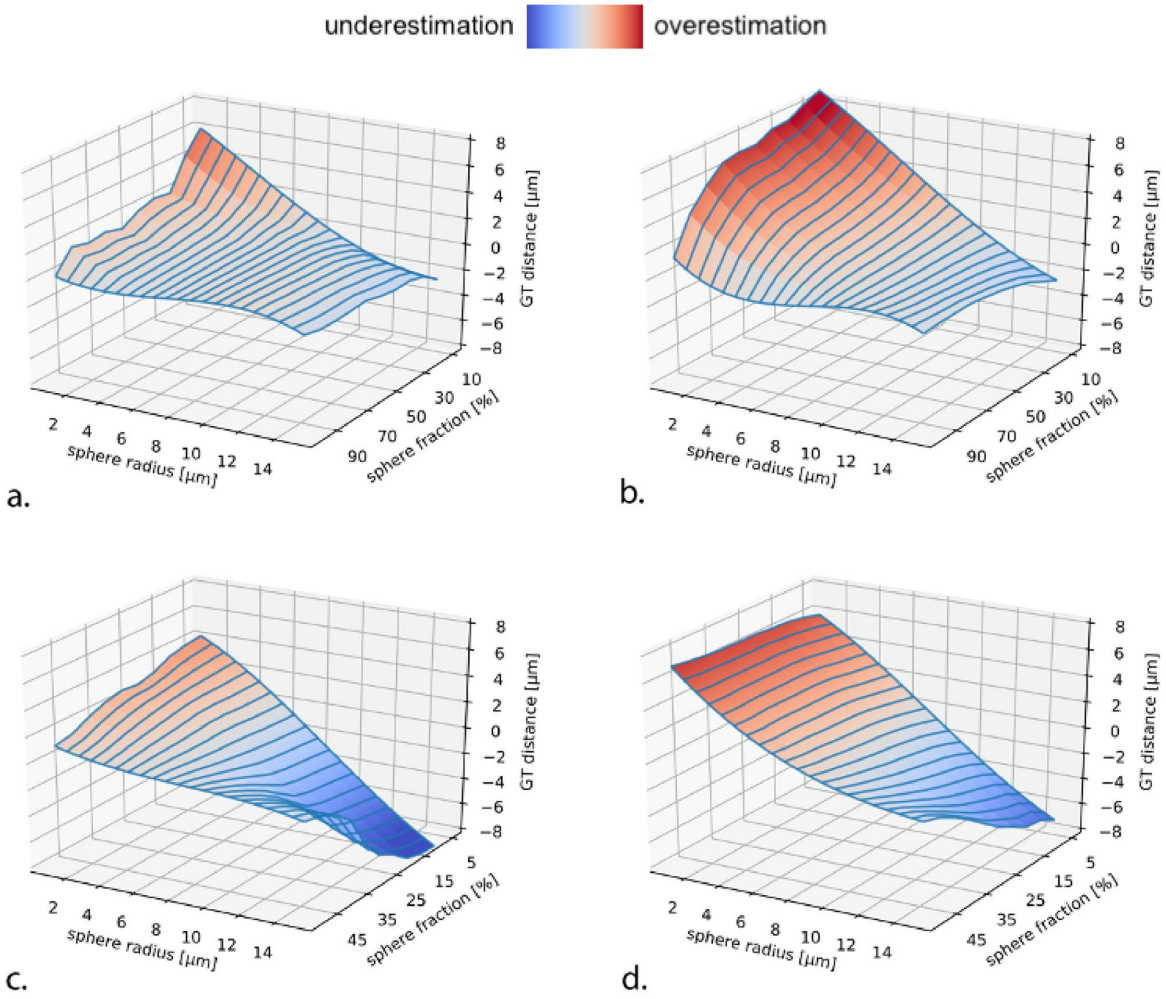
Numerical simulations show the accuracy of sphere radius estimates in the CONNECTOM scanner (**a,c**) and on a clinical scanner (**b,d**). (**a,b**) “Intra-cellular”: we included spheres, simulating cells, and sticks, simulating neurites, in equal proportion. (**c,d**) “Intra-cellular and extra-cellular”: here, we added an extra axonal signal simulated with tensor components. In all simulations, Rician noise with SNR = 50 was added. Distance (defined as difference in μm) from the ground truth (GT) is evaluated. Red = overestimation, blue = underestimation, light = acceptable error. Simulation parameters can be found in the “Numerical simulation” in “Methods” section.

Extensive numerical simulations were performed to compare the accuracy results in two different diffusion protocols. The results are shown in Fig. 1.

The sphere compartment is quantified using the soma fraction (*f*_*soma*_) estimated with SANDI.

The results of the “intra-cellular” simulation show that—using the CONNECTOM scanner—the estimates provided by SANDI are correct for a sphere radius larger than 8 μm (Fig. 1a). Furthermore, it is possible to recover a sphere radius ≥ 2 μm if the sphere density is 100% (Fig. 1a). When simulating the protocol of a Prisma scanner, the sphere radius is correctly estimated when it is larger than 10 μm, whereas there is an overestimation for a sphere radius lower than 10 μm (Fig. 1b). In this scenario, it is possible to recover a sphere with a radius of 6 μm if the density of spheres is close to 100%; similarly, it is possible to estimate a sphere radius of 8 μm with a sphere density of 50%; radii lower than 6 μm are overestimated.

Figure 1c,d show the “intra-cellular and extra-cellular” simulation*.* Here, we added some complexity to the microstructure substrate, i.e., an extra axonal signal simulated with tensor components. In this case, the estimates increase in uncertainty. Even using the CONNECTOM scanner, at least 40% of the sphere’s density is necessary for the signal to retrieve the sphere radius correctly (Fig. 1c). When the sphere density is lower than 40%, we overestimate spheres with a radius smaller than 6 μm and underestimate spheres with a radius larger than 6 μm. In the Prisma scanner, Fig. 1d, we need at least 50% of the sphere’s density to retrieve the sphere radius correctly for spheres with a radius larger than 10 μm. Spheres smaller than 10 μm cannot be recovered.

### Cortical soma fraction in multiple sclerosis patients and healthy controls

**Figure 2:**
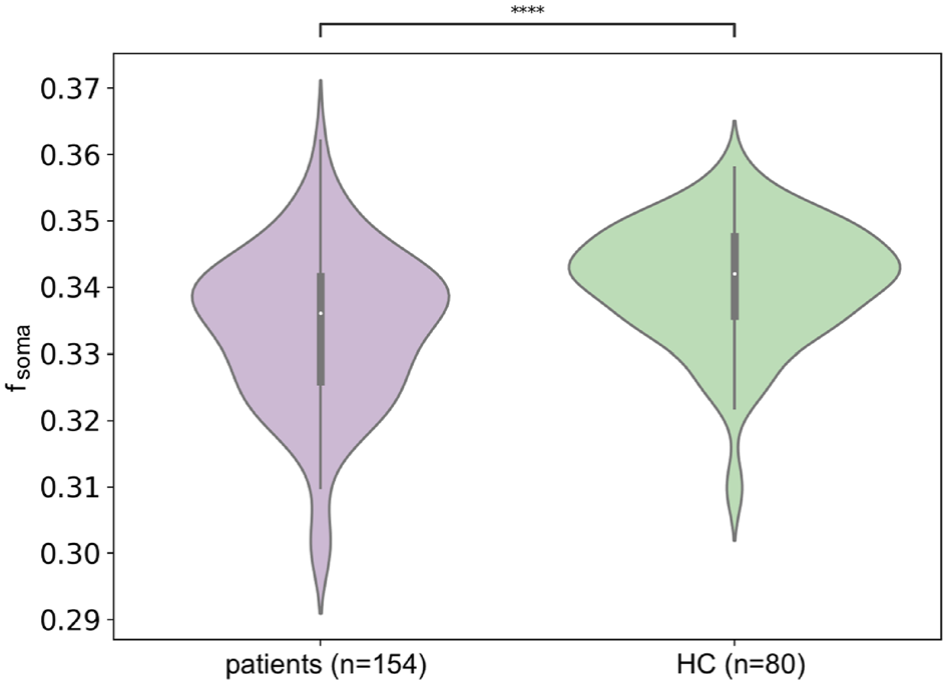
Boxplot of the comparison of the soma fraction (f_soma_) index in the whole cortex between MS patients vs. healthy controls (HC).

In Fig. 2, we report the boxplot comparing the *f*_*soma*_ estimation in MS patients vs. healthy controls (HC). The mean *f*_*soma*_ is lower in MS patients’ cortex than in HC (*f*_*soma*_ patients 0.33 ± 0.02 and HC 0.34 ± 0.01, t-statistic: 4.19, p-value < 0.0001).

**Figure 3:**
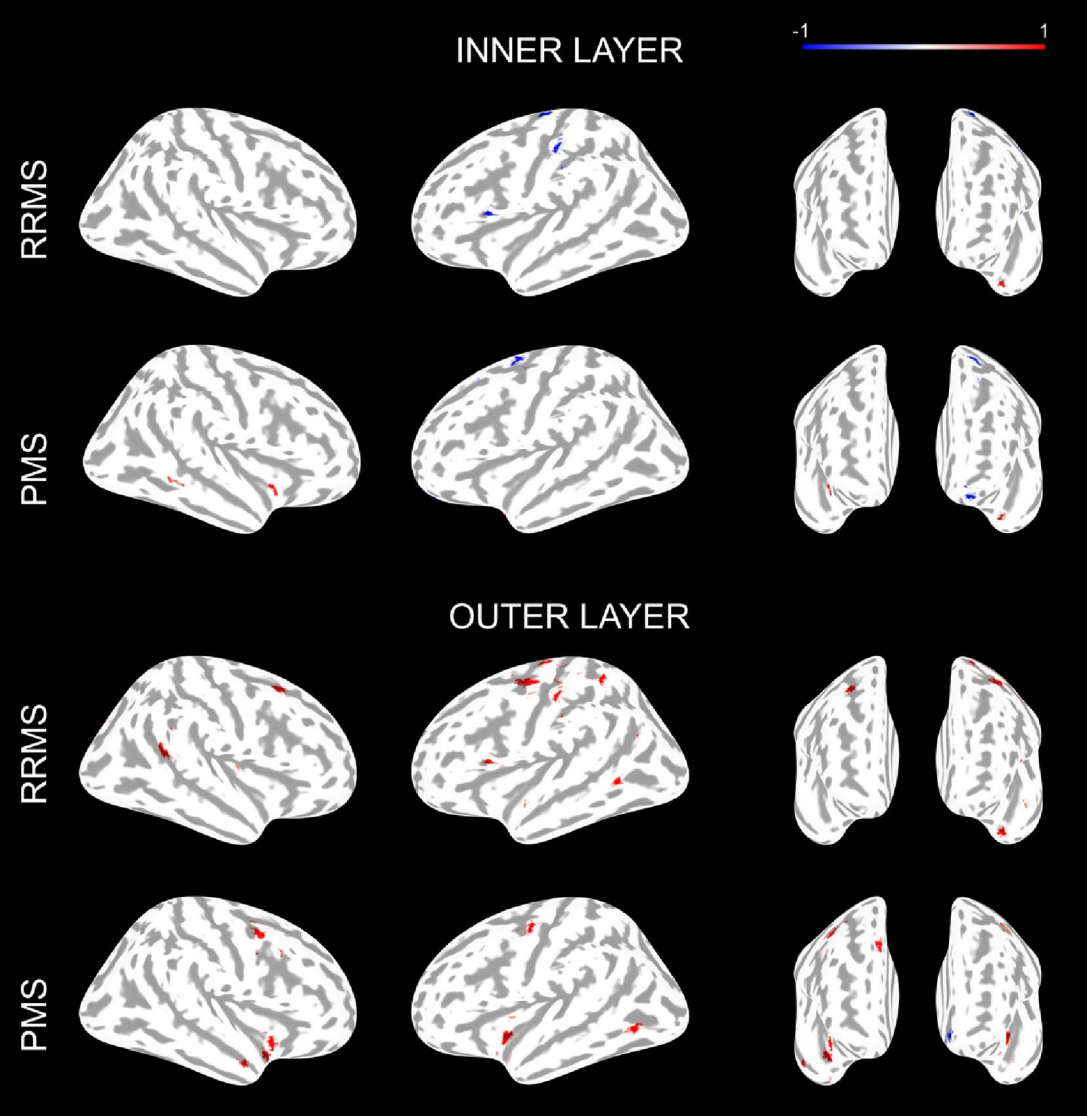
Results of the general linear model group analysis performed with Freesurfer between RRMS vs. HC and PMS vs. HC for f_soma_ parameter. The analysis divided the cortex into inner and outer layers using 50% of the cortical depth. Details of all clusters are in Supplementary Table 1.

When we compared the average *f*_*soma*_ in the outer and inner cortical layer between MS patients and HC, however, we observed that patients exhibited predominantly decreased *f*_*soma*_ in the inner layer of the cortex in MS patients vs. HC (p < 0.05, Fig. 3). In RRMS patients, we found four clusters with decreased *f*_*soma*_ compared to HC (total size: 100.65 mm^2^ (p < 0.03); one cluster in the temporal pole showed increased *f*_*soma*_ in RRMS vs. HC (size: 41.79 mm^2^, p = 0.002). In PMS, we also found four clusters with decreased *f*_*soma*_ (total size: 116.46, p < 0.03) and one cluster in the superior temporal area (size: 23.07 mm^2^, p = 0.035).

The cortex’ outer layer showed predominantly increased *f*_*soma*_ compared to controls (p < 0.05, Fig. 3). Five clusters of increased *f*_*soma*_ were found in relapsing–remitting MS patients (RRMS) vs. HC (p = 0.0002), for a total size of 221.6 mm^2^, with the biggest cluster located in the precentral area of the left hemisphere (89.34 mm^2^). Ten clusters of increased *f*_*soma*_ were found in progressive MS patients (PMS) vs. HC (p = 0.0002), for a total size of 296.08 mm^2^ (p < 0.05), and with the most extended cluster located in the right hemisphere in the caudal middle frontal area (54.48 mm^2^). In the outer layer of the cortex, we also found a few clusters where the *f*_*soma*_ was decreased in MS patients vs. HC (p < 0.05). RRMS had 3 clusters of decreased *f*_*soma*_ compared to HC (total size of 64.91 mm^2^, p < 0.05); in PMS only one cluster located in the medial orbitofrontal area exhibited decreased *f*_*soma*_ compared to HC (total size: 22.81 mm^2^, p = 0.0339).

### Comparison of soma fraction in cortical lesions between RRMS and PMS patients

**Figure 4:**
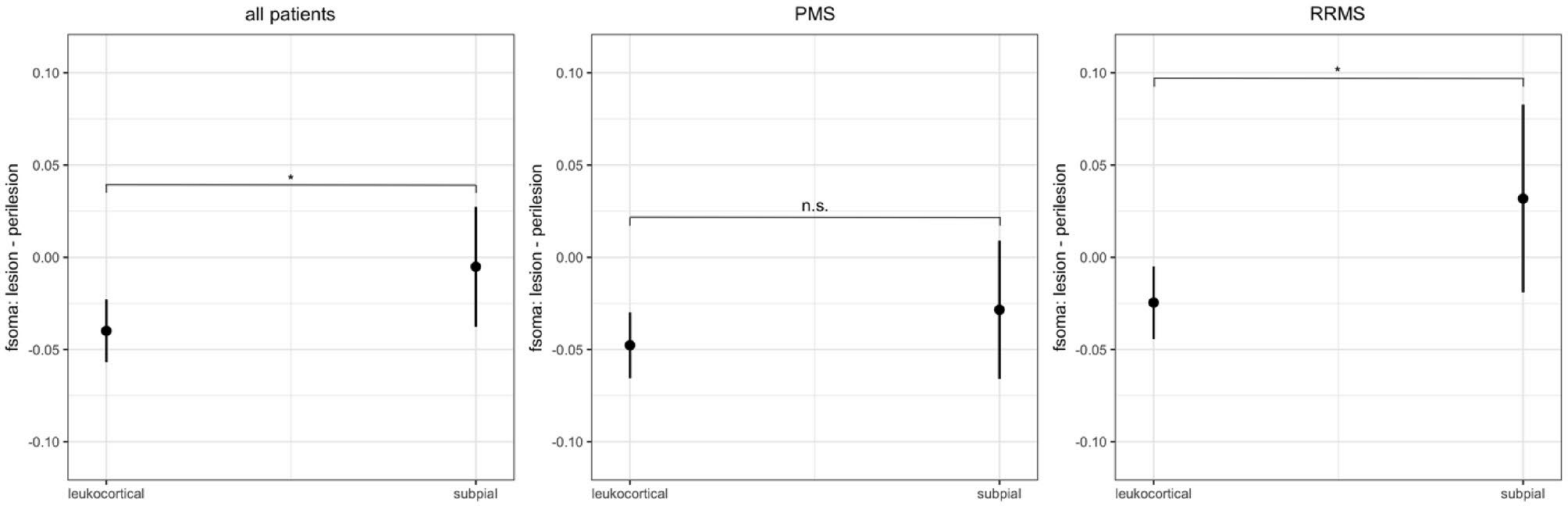
The left plot shows the difference between the Δ f_soma_ (lesion—perilesion tissue), defined as the difference between the mean f_soma_ in the lesion voxels minus the mean f_soma_ in the perilesional voxels, by lesion type across all patients, i.e., leukocortical and subpial. The center and the right plot show the delta mean f_soma_ in the lesion and perilesion, respectively, in PMS and RRMS. Error bars in the plots are 95% confidence intervals.

When we calculated the difference between the mean *f*_*soma*_ in lesions (subpial and the grey matter part of leukocortical) and the mean *f*_*soma*_ in the corresponding perilesional tissue (delta mean), see Fig. 4, we found that RRMS patients had higher delta *f*_*soma*_ in subpial lesions compared to leukocortical lesions whereas PMS patients did not (RRMS t-statistic: 2.249, p = 0.026; PMS t-statistic: 1.075, p = 0.284).

### Correlation of f_soma_ with quantitative MRI parameters in cortical lesions

**Figure 5:**
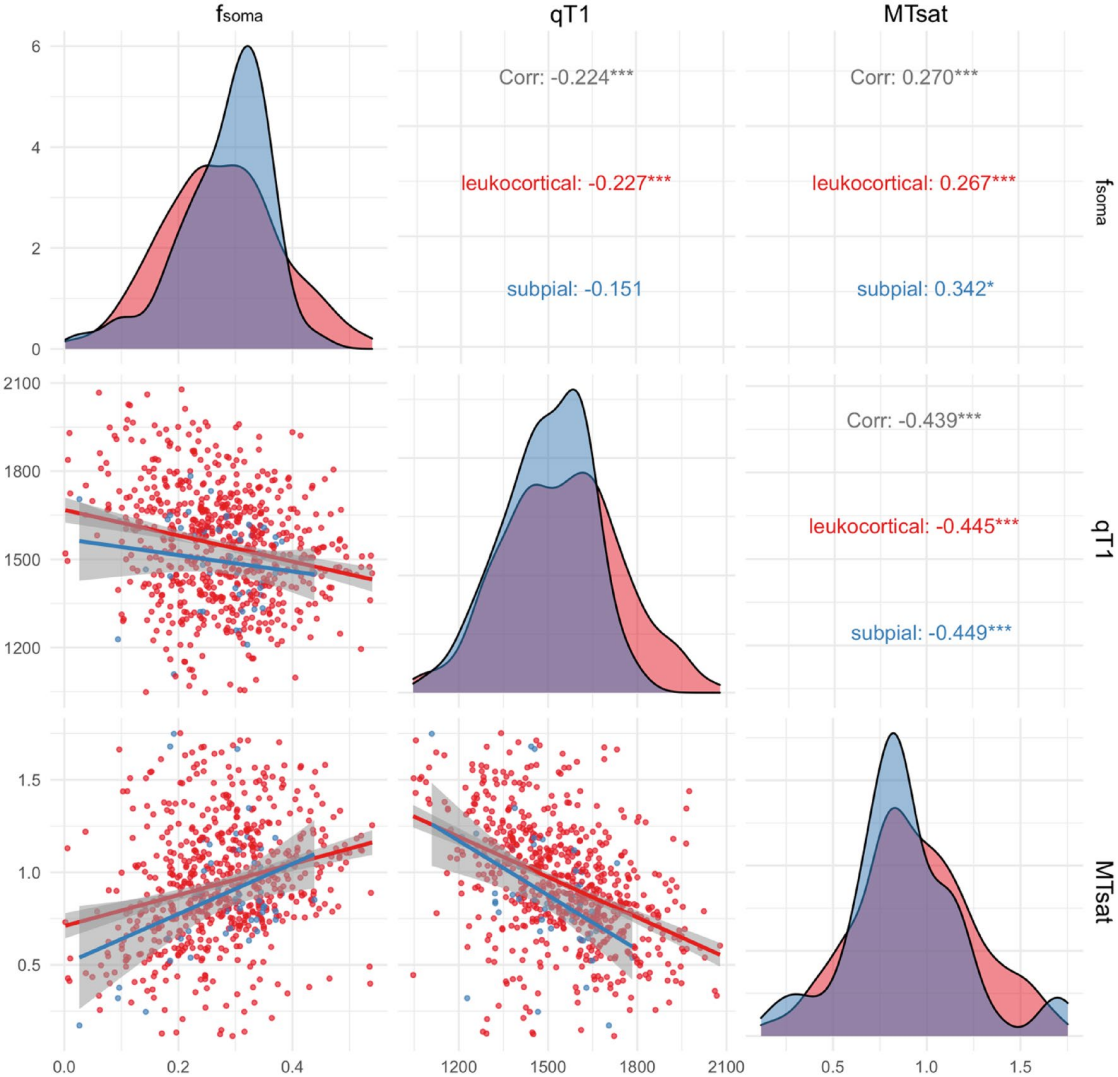
Correlation of f_soma_ with quantitative T1(qT1) and magnetization transfer saturation (MTsat) in subpial (blue) and leukocortical (red) lesions. ‘*’p < 0.05, ‘**’p < 0.01, ‘***’p < 0.001. Error band in the plots are 95% confidence intervals.

The *f*_*soma*_ was negatively with quantitative T1 (qT1), which *increase* is associated with tissue loss, which can be due to a range of pathological processes including gross tissue loss, complete tissue loss; and positively related to Magnetization Transfer saturation (MTsat), which *decrease* is associated with demyelination, axonal injury, or other forms of cellular damage, for both lesion groups (Fig. 5).

### Correlation between cortical soma fraction and clinical scores

No significant correlation was found between cortical cellular fraction and Expanded Disability Status Scale (EDSS), Multiple Sclerosis Severity Score (MSSS), and Symbol Digit Modalities Test (SDMT) z-scores.

### Correlation between cortical soma fraction and serum neuro-filament light chain

**Figure 6:**
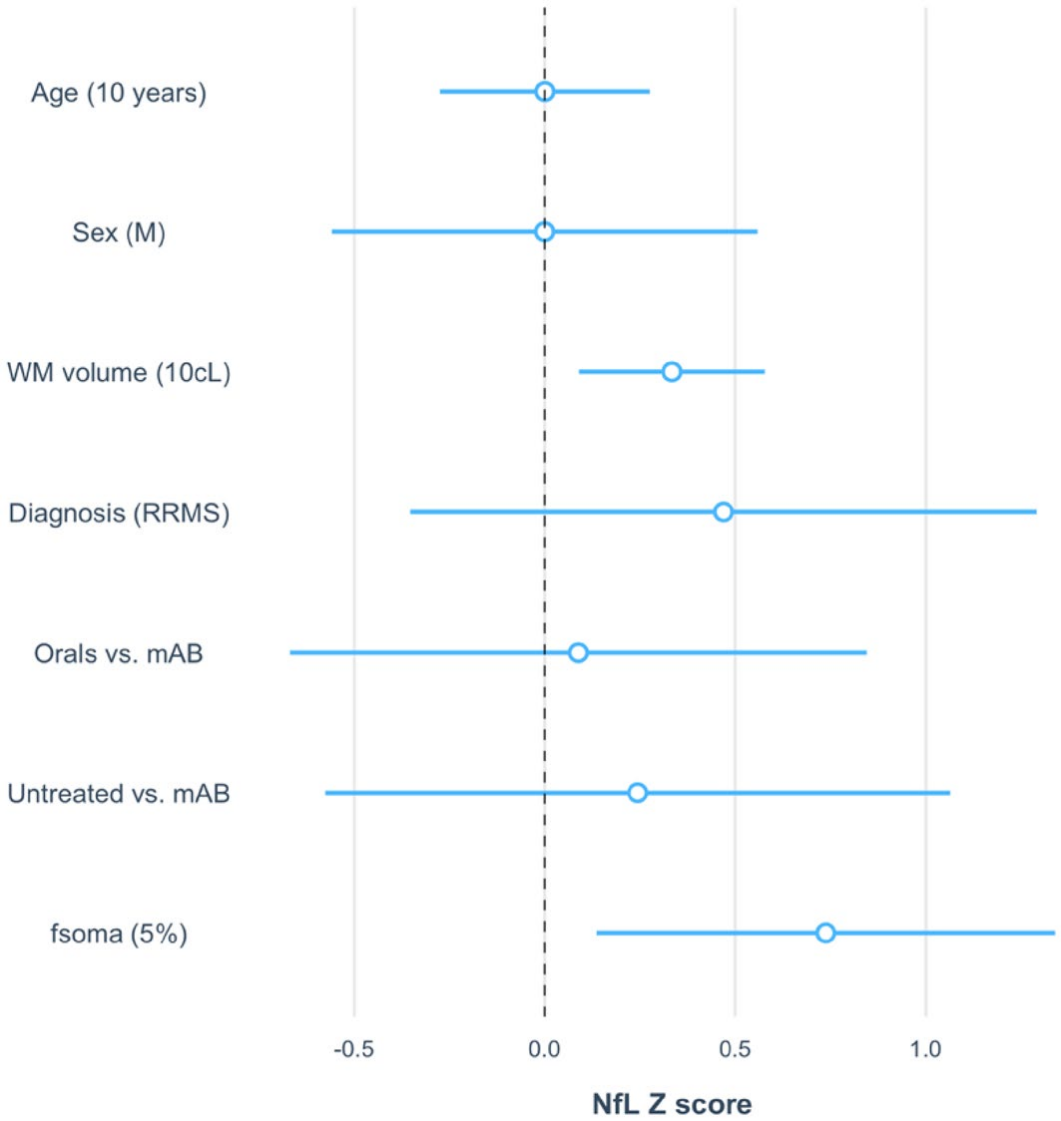
Linear model assessing the relationship between f_soma_ and age-corrected sNfL z-scores. Abbreviations: male (M), white matter (WM), centiliter (cl), Relapsing–remitting MS (RRMS), monoclonal antibody (mAB). Point estimates and 95% confidence intervals.

Interestingly, we found that both WM lesion volume (p < 0.001, estimate: 0.334) and mean *f*_*soma*_ in cortical lesions (p < 0.05, estimate: 0.738) explained the increase in age-corrected^43^ serum neurofilament light chain (sNfL) levels in MS patients. Both WM lesion volume (p = 0.008, est = 0.334) and *f*_*soma*_ (p = 0.017, estimate = 0.738) were significantly and positively related to an increase in sNfL z-scores, see Fig. 6 (for details, see Supplementary Table 2).

The model included SANDI *f*_*soma*_ (unit: 5% increase) other co-variates known to influence sNfL, such as age (unit: 10 years), WM lesion volume (unit: 10 cl), MS subtype (RRMS/PMS), and treatment. In this model, NfL z-scores were used as described in Benkert et al.^43^. In short, sNfL z-scores are measures of sNfL deviations from a normal population, which take into consideration a patient’s age and body mass index (BMI).

Additionally, we have performed a partial correlation analysis to determine the unique contribution of *f*_*soma*_ to the variance in sNfL levels, independent of white matter lesion load. The inclusion of *f*_*soma*_ results in an Adjusted R-squared of 0.1088, while the model without *f*_*soma*_ has an Adjusted R-squared of 0.001492. This indicates that *f*_*soma*_ explains an additional 10.74% of the variance in sNfL. The likelihood ratio test comparing the full and reduced models yields a p-value of 0.01413, which is statistically significant.

## Discussion

This work showed that the application of SANDI to DW-MRI data acquired in a clinical 3T scanner enhances our ability to characterize cortical pathology in pwMS. Using this approach, in fact, we provided first evidence that cortical changes in SANDI f_soma_—an indirect measure of tissue cellularity—are compatible with inflammatory and degenerative phenomena in the cortex of pwMS, which were previously only shown in histopathological studies^44^.

We applied SANDI to a DW-MRI protocol acquired in a 3T clinical scanner, which did not allow us to model potential diffusion–exchange between tissue compartments. Moreover, we judged best not to introduce constraints to the model, in order not to bias its estimates in pathological tissue. Because of those conditions and assumptions, we applied numerical simulation to assess the performance of SANDI to estimate the parameter *f*_*soma*_. Our data showed that even when a large component of extra-cellular water is included in the model, we can reliably measure only spheres with a radius of 8 μm in a condition with a large amount of density of spheres (50%): this corresponds to a diameter of 16 μm, which may be found in densely cellular tissue such as layers I–III or in inflammatory areas where large cells are recruited (e.g., subpial lesions)^45^.

The original implementation of SANDI^39^ was performed using a more advanced DW-MRI data acquired in a research-only CONNECTOM system, i.e., a scanner with significantly more powerful diffusion gradient strengths than the one applied in this study. The previous study aimed at determining the density and distribution of soma sizes in each voxel. They utilized the CONNECTOM system with the SANDI model to achieve a maximal resolution of a *f*_*soma*_ sphere of 2 μm radius. However, to apply the SANDI model to a clinical DW-MRI protocol, we simplified the AMICO-SANDI^46^ dictionary. This simplification was achieved by reducing the number of parameters required to estimate the model. Specifically, the sphere diffusivities were fixed to a single value for both neurite and isotropic-restricted compartmets and three values for extra-cellular compartment. Moreover, the radii of spheres to estimate were limited in a range achievable in a clinical MRI system with 80 mT/m of diffusion gradient strength, resulting in a reduction in the granularity of soma size estimation.

When we applied the SANDI model to investigate the cortical *f*_*soma*_ characteristics in a large cohort of MS patients and healthy controls, we found that pwMS exhibited a significant global decrease in *f*_*soma*._ These results confirmed previous findings obtained in a small cohort of 23 pwMS and 20 healthy subjects^41^, where the original SANDI model was applied to data acquired in a clinical 3T scanner. Different than our study, however, this previous work^41^ applied SANDI to data acquired with a lower spatial resolution (2.5 mm vs. 1.8 mm isotropic), which prevented the analysis of *f*_*soma*_ variations in different cortical layers as well as in cortical lesions.

We investigated the *f*_*soma*_ behavior in the outer part of the cortex (where subpial demyelination usually occurs) and in the inner part of the cortex, where leukocortical lesions are mostly located^47^. Interestingly, we found that the outer part of the cortex in MS patients exhibited localized increase in *f*_*soma*_ compared to healthy subjects, especially in progressive MS patients. These increases were not uniform across the entire cortex but were rather region-specific. Extensive subpial demyelination in MS patients has been shown in many postmortem studies^44,45^, which also evidenced the accumulation of reactive microglia cells in the outer layers of the cortex^15^. Since activated microglia cells have a round shape^48^ and a mean maximal diameter^48^ of 14.6 μm, it is plausible that *f*_*soma*_ is sensitive to their local accumulation in vivo in MS patients.

The prevalence of more extensive demyelination in the outer layers of the cortex has been shown in several previous studies (Pardini et al.^49^ and Mainero et al.^50^); nevertheless, up to the present time, there has been no means for quantifying changes in cellularity-related parameters within the cortex.

**Figure 7:**
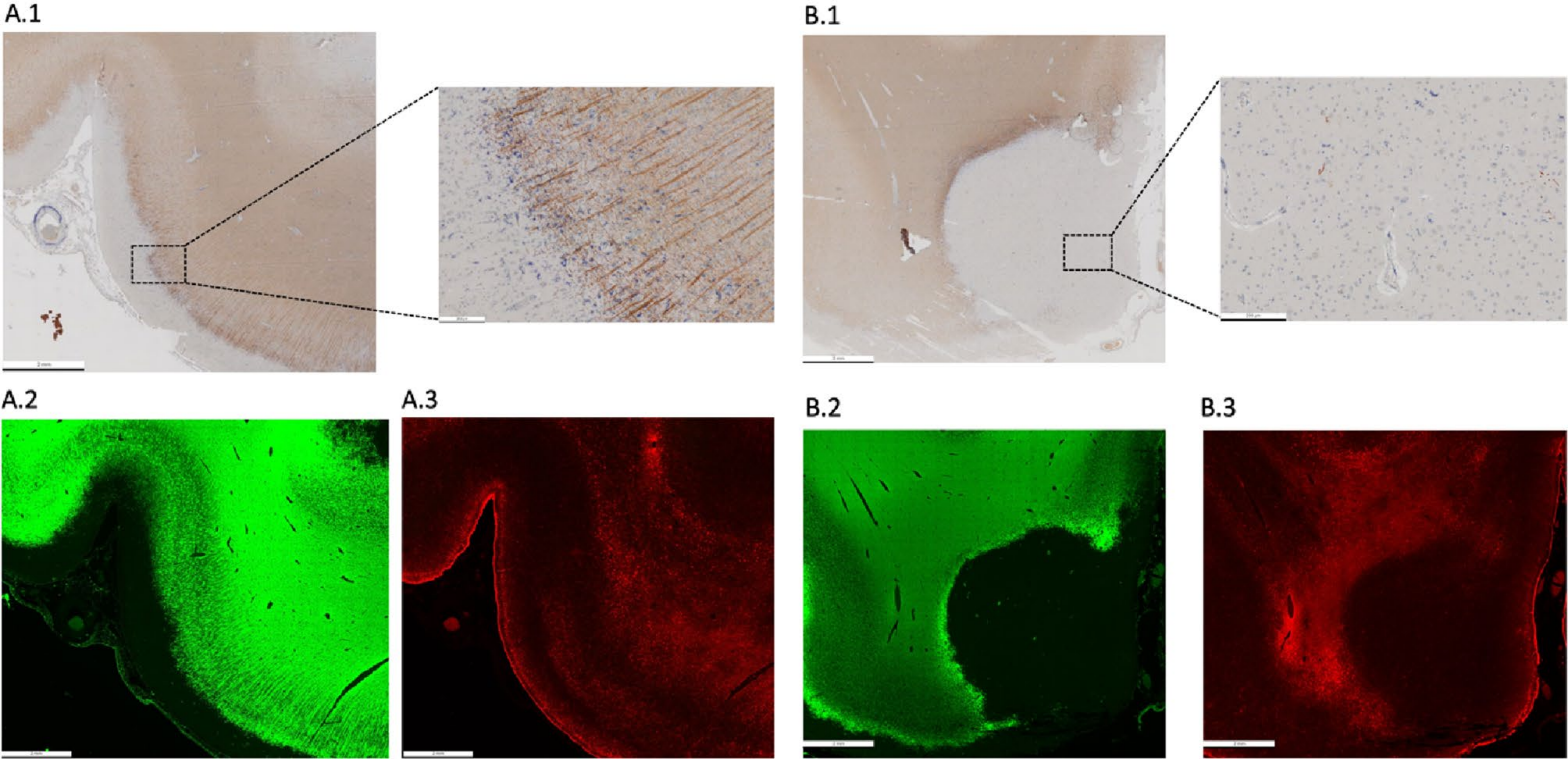
Histopathology of one subpial and one leukocortical lesion. (**A**) Myelin basic protein (MBP) immunohistochemical staining (**A.1**) shows a subpial lesion with microglia accumulation in the central cortical layers. Double immunofluorescence immunohistochemical staining for myelin (MBP, **A.2**) and astrocytes (Glial Fibrillary Acidic Protein—GFAP-, **A.3**) shows only partial depletion of astrocytes. (**B**) Myelin basic protein (MBP) immunohistochemical staining (**B.1**) shows a leukocortical lesion where microglia is absent in its grey matter part and present to a certain extent only in its white matter part. Double immunofluorescence immunohistochemical staining for myelin (MBP, **B.2**) and astrocytes (Glial Fibrillary Acidic Protein—GFAP-, **B.3**) shows a complete depletion of astrocytes.

Figure 7 is included in the manuscript for illustrative purposes only. The examples depicted in the figure are representative images that are intended to visually demonstrate the appearance of subpial and leukocortical lesions with different stainings. These images are provided to aid the reader’s understanding the characteristics of those specific lesions. In particular, Fig. 7 presents two examples of subpial and leukocortical lesions with different stainings: first, a subpial lesion (Fig. 7A) with an accumulation of microglia in mid-cortex (Fig. 7A.1,A.3); second, a leukocortical lesion (Fig. 7B) with accumulated microglia at the border and complete depletion of astrocytes (Fig. 7B.3).

Our MRI analysis showed an increased *f*_*soma*_ in subpial lesions compared to the grey matter part of leukocortical lesions in relapsing–remitting MS patients, suggesting increased cellularity in subpial plaques in this patients group. The partial presence of astrocytes and activated innate inflammatory cells (microglia and macrophages)^51^ in subpial lesions has already been shown in postmortem studies^52–54^ as well as in a work applying ^11^C-PBR28 positron emission tomography (PET) in MS patients^29^. Our study contributes to this body of evidence by suggesting that SANDI may offer a non-invasive means to quantify such changes in vivo.

On the other hand, we saw a reduction SANDI *f*_*soma*_ in the inner part of the cortex in pwMS compared to HC, which is a region that spatially corresponds to layers IV and V, where pyramidal neurons are located. As a consequence, the measured decrease in *f*_*soma*_ might well reflect neuronal degeneration due to Wallerian degenerative phenomena originating from WM lesions^55^ or local mechanisms within the cortex, leading to neuronal loss^56^.

Our data may suggest that it is possible to measure ongoing degenerative and inflammatory processes in the cortex of pwMS. These findings are particularly important because the subpial component of cortical injury is related to the development of progressive disease in pwMS^10,24^; the possibility to quantify ongoing inflammatory/neurodegenerative events in the cortex of MS patients might therefore open the perspective to assess new disease-modifying treatments targeting the cortex.

To assess the relationship between SANDI *f*_*soma*_ and other microstructural tissue properties measures, we correlated SANDI *f*_*soma*_ with global measures of microstructural loss (quantitative T1), and of myelin/cellular damage^57,58^ (MTsat). The *f*_*soma*_ positively correlated to MTsat and negatively to qT1. MTsat is a quantitative MRI parameter that reflects the exchange of magnetization between free water protons and protons bound to macromolecules, such as those found in cellular membranes. Higher MTsat values typically indicate a greater presence of macromolecular structures, which can be associated with increased cellular density. The positive correlation between *f*_*soma*_ and MTsat may therefore suggest that regions with a higher density or size of cell bodies also have a higher content of macromolecules, which could be due to cellular components. On the other hand, quantitative T1 is a measure of the longitudinal relaxation time of tissue and is sensitive to microstructural loss. Higher qT1 values typically indicate greater tissue damage or loss, which could be due to factors such as axonal loss, or other pathological changes. The negative correlation between f soma and qT1 suggests that areas with higher cell body density (higher f soma) tend to have less microstructural loss (lower qT1), and vice versa. This relationship may reflect the fact that areas with ongoing neuroinflammatory processes or neurodegeneration, which would be indicated by a decrease in cell body density, also show increased microstructural loss as captured by qT1 measurements.

We did not find statistical significance in *f*_*soma*_ when used to predict EDSS, MSSS, and SDMT. These measures are in fact reflecting a global burden of MS pathology, not only cortical damage. However, we found that an increase in *f*_*soma*_ was related to an increase in sNfL in MS patients^59^. Higher levels of NfL in both CSF and blood have consistently been associated with ongoing inflammatory phenomena^60^, suggesting again that *f*_*soma*_ is a parameter that is sensitive to ongoing inflammatory processes.

### Limitations

Different factors could cause soma size and density to increase or decrease, there are several biological and pathological processes that can influence these changes: MS can lead to neuronal loss and atrophy, which would manifest as a decrease in soma size and density; neurons can undergo structural changes in response to learning, memory formation, or environmental stimuli, which can lead to changes in soma size; inflammatory processes in the brain can lead to changes in soma size and density, either through direct effects on neurons or indirectly through effects on glial cell (e.g. activation); exposure to neurotoxins or drugs can lead to neuronal damage or death, affecting soma size and density; certain infections can lead to neuronal damage, which can affect soma size and density; normal aging processes can lead to changes in neuronal structure and function, including changes in soma size and density.

Furthermore, the effects of various pathological and physiological changes on SANDI estimates can be complex, as each condition may alter the diffusion properties of water in the brain in different ways: the presence of *edema*, which increases the extracellular space, can lead to an apparent increase in the extracellular volume fraction and a decrease in the intracellular volume fraction, this could potentially be interpreted by the SANDI model as a decrease in cell density or soma size, conversely, the resolution of edema would likely reverse these changes; *cellular swelling* (cytotoxic edema) can lead to a reduction in the extracellular space and an increase in the intracellular volume fraction, this might be reflected in SANDI estimates as an increase in soma size or density, depending on the extent of the swelling and the specificities of the model’s interpretation of the diffusion signal; *loss of neurites*, as seen in neurodegenerative diseases or acute injury, would likely result in a decrease in the neurite density estimate from SANDI, recovery or regeneration of neurites could increase the neurite density estimate; *neuronal loss* would be expected to decrease the soma density estimate from SANDI, as there would be fewer cell bodies contributing to the intracellular volume fraction; *demyelination* primarily affects the white matter, but it can also influence the diffusion properties in gray matter due to changes in the extracellular space and potential secondary effects on neurons, in the context of SANDI, demyelination could alter the diffusion signal, potentially affecting the estimates of soma and neurite density; *inflammation* can cause a variety of changes, including edema and cellular infiltration, which can affect the diffusion properties of the tissue, SANDI estimates might reflect these changes as alterations in soma and neurite density, but the specific effects would depend on the nature and extent of the inflammation.

Additionally, areas with significant tissue loss may change tissue density due to the collapse of surrounding tissue into spaces formerly occupied by water-filled intracellular components. In our study, we observed that *f*_*soma*_, increased in the outer part of the cortex and in subpial lesions. This could potentially be interpreted as an increase in tissue density resulting from the collapse of the extracellular matrix and the compaction of cellular elements as a consequence of atrophy. However, it is also important to consider that increased *f*_*soma*_ could be indicative of inflammatory processes, as suggested by the positive correlation between fsoma and sNfL, a marker that is very sensitive to neuroinflammation, in MS patients. Without direct histopathological correlation, it is challenging to ascertain the precise nature of these changes.

We could not assess a broad spectrum of *f*_*soma*_ characteristics, since those become evident only when b-values ~ 5000 and 10,000 are reached^39^, which was partially achievable only in the setting proposed in the study Margoni et al.^41^.

In addition, the applied SANDI model did not consider the presence of intercompartment water exchange^61^, a feature that must be implemented in future models to increase the sensitivity to lower densities of spheric structures^62,63^, which might be especially beneficial in lesions. Furthermore, multiple diffusion econding^63^ might be applied in the future to limit the problem of degeneration of the estimation of SANDI parameters.

Partial volume effects can be particularly problematic in the cortex due to its highly folded nature and the presence of cerebrospinal fluid (CSF) in the sulci. These effects can lead to an underestimation or overestimation of cortical thickness and may affect the measurement of other cortical properties, such as tissue density or microstructural integrity. We can not exclude that our findings are biased by CSF contamination in the cortex, although this should have rather limited the observed increase of *f*_*soma*_ than lead to erroneous results. To avoid this incertitude, future work may apply FLAIR prepared DW-MRI^64^ acquisition to remove the CSF signal or use the free-water elimination processing step to mitigate it.

Regarding the “inner cortical” measurements, which are intended to approximate the location of the inner cortex, will likely include signal from both the outer cortex and adjacent WM. Similarly, measurements attributed to the “outer cortex” may also include signal from the CSF in the sulci or from the inner cortical layers. These partial volume effects are indeed exacerbated by cortical thinning, which is a prominent feature of MS, particularly in its progressive forms, and can be associated with the presence of cortical lesions.

It is important to note that while perilesional tissue is not entirely normal and may contain some degree of pathology, it is still a more appropriate reference than distant normal-appearing gray matter, as it accounts for regional variations in the cortex that could affect the interpretation of lesional changes. Moreover, we acknowledge that the perilesional tissue of subpial and leukocortical lesions may exhibit different characteristics, which could potentially influence the comparison of deltas.

Furthermore, in this work we used MP2RAGE and not MPRAGE for the detection of subpial lesions: MP2RAGE offers, in fact, an increased sensitivity to cortical focal pathology than MPRAGE^65,66^. Among cortical lesions, subpial lesions are challenging to detect using the currently available MRI method.

Nevertheless, MP2RAGE appears to be more sensitive to the presence of subpial lesions than other advanced MRI sequences used to detect them, such as Double Inversion Recovery (DIR) and hase-sensitive inversion recovery (PSIR)^66^. Yet, also MP2RAGE appears to detect only a minority (ca 5%) of focal subpial demyelination, highlighting the challenge in detecting these lesions with non-invasive approaches^66^.

In addition, although the simulations utilized are advanced, they are still simplified versions of the complex in vivo environment. More complex simulations should be performed in the future. Additionally, if histological and MRI tests were done in tandem on the same MS samples, this would allow for a more accurate calibration of the SANDI technique and a better understanding of its effectiveness in different tissue settings.

We acknowledge that the assumptions and constraints within the SANDI model itself can influence the estimates. For example, assigning a single numerical value for the diffusivity of neurite and isotropic-restricted compartments is a simplification that may not fully account for the diverse properties of biological tissues. Advanced models could incorporate various diffusivity values that more accurately depict the diversity of neural tissue and the intricate relationships between different cellular structures. Furthermore, in the clinical application of SANDI, certain constraints were not introduced to avoid biasing the estimates in pathological tissue, which could affect the granularity of soma size estimation. SANDI provides a mean value per voxel, which represents an average over the distribution of cell sizes within that voxel. While it is possible to extract distributions of different sizes, the stability and reliability of these measurements need to be thoroughly validated before drawing definitive conclusions. Some work in this direction has been conducted by Romascano et al.^67^ where they estimated distributions of axon diameters using diffusion MRI. Their approach to estimating microstructural features from diffusion MRI data can serve as a reference for our future studies on soma size and density estimation using SANDI.

In addition to the points already discussed, it is worth noting that DW-MRI can serve as an indirect indicator of cellularity and that it can potentially deduce the presence and quantity of particular cell types, such as microglia, macrophages and potentially astocytes, rather than directly observing them.

## Conclusion

In conclusion, we present a novel methodology to study “cellularity-related” changes in-vivo in MS patients. Using this method, we found that pwMS had an overall decrease in cellularity (*f*_*soma*_*)* in the cortex, which is compatible with global cortical degeneration. Furthermore, progressive MS patients had higher *f*_*soma*_ in the outer layer of the cortex and relapsing–remitting patients had increased *f*_*soma*_ in subpial lesions, which is compatible with pathologically-known inflammatory phenomena in these areas. The *f*_*soma*_ parameter was also significantly correlated with sNfL, a marker of inflammatory axonal damage. Overall, our data show that SANDI *f*_*soma*_ is a very promising biomarker to monitor changes in cellularity compatible with neurodegeneration and neuroinflammation in the cortex of MS patients.

## Methods

### Participants

We enrolled a total of 154 multiple sclerosis patients, 98 with relapsing–remitting MS (RRMS), 23 with primary-progressive MS (PPMS), 33 with secondary-progressive MS (SPMS), and 80 healthy controls (Table 1). The inclusion criteria were: (i) multiple sclerosis diagnosis according to McDonald criteria 2018^68^ and diagnosis of active RRMS or inactive PMS as defined by Lublin et al.^69^; (ii) absence of any concomitant psychiatric or neurological disease (excluding headache); and (iii) absence of contraindication to MRI. Exclusion criteria were pregnancy, contraindication (e.g., claustrophobia, pacemaker) to magnetic resonance imaging (MRI), and inability to give informed consent. The ethics review committee of the University Hospital Basel (IRB of Northwest Switzerland) approved the study, and all participants entered the study following written informed consent. All research was performed in accordance with relevant guidelines/regulations.

**Table 1:** Patient and control demographics.

	Multiple sclerosis	Healthy controls
Sex, n (male/female)	155 (62/93)	80 (33/47)
Age, years, mean ± SD	46 ± 15	37 ± 13
EDSS score, median (range)	2.5 (0–8)	–
Disease course, RR/PMS	98/57	–

*SD* standard deviation.

### MRI acquisition

MRI was performed on a 3T whole-body magnetic resonance system (Magnetom Prisma, Siemens Healthineers, Erlangen, Germany) using a 64-channel phased-array head and neck coil for radio frequency reception. The MRI protocols included: (i) 3D FLAIR (repetition time/echo time/inversion time = 5000/386/1800 ms), acquisition time 5 min 40 s with 1 mm isotropic spatial resolution (ii) MP2RAGE (repetition time/inversion time 1/inversion time 2 = 5000/700/2500 ms), acquisition time 8 min 20 s with 1 mm isotropic spatial resolution was used to calculate quantitative T1 (qT1)^65^; (iii) magnetization transfer weighted (MTw), T1 weighted (T1w), and proton density-weighted (PDw) FLASH sequences, total acquisition time 7 min 54 s with 1.33 mm isotropic spatial resolution as well as a B1 excitation field mapping was used to calculate Magnetization Transfer saturation MTsat^70,71^; (v) DW-MRI (repetition time/echo time/δ/Δ/resolution = 4.5 s/75 ms/19 ms/36 ms/1.8 mm) isotropic with b-values 0/700/1000/2000/3000 s/mm^2^ with 12/6/20/45/66 measurements, respectively, per shell and 12 measurements of b-value 0 s/mm^2^ with reversed phase encoding, total acquisition time 15 min 30 s.

### Lesion segmentation

Automatic segmentation of white matter and cortical lesions was performed using a deep-learning-based method^72^. The algorithm approach consisted of a cascade of two convolutional neural networks and was adjusted to take as input FLAIR and MP2RAGE MRI contrasts. Manual correction of automatic white matter lesion masks was performed on FLAIR. For the cortical lesions, after the intial automatic segmentation, two experienced readers manually corrected the automatic output of the lesions on MP2RAGE by consensus, and cortical lesions were divided into subpial and leukocortical lesions^73^. 8199 white matter lesions (mean number 52.9, std 42.0 per patient) segmented and found in all 154 patients and 726 cortical lesions (mean number 8.7, std 11.3 per patient) were segmented and found only in 98 patients. Cortical lesions were classified into 51 being subpial and 675 being leukocortical. All lesion masks were co-registered in the DW-MRI space. Peri-lesion masks were calculated as one voxel dilation (in the DW-MRI space) around the lesion.

### Clinical assessment

Multiple sclerosis disability was assessed for all patients using Neurostatus-EDSS (www.neurostatus.net)^74^ by certified neurologists at University Hospital Basel.

The multiple sclerosis severity score (MSSS) was calculated by combining the expanded disability status scale (EDSS) and disease duration^75^.

### Cognitive assessment

Forty-two patients, all of them with cortical lesions, underwent neuropsychological examination with the symbol digit modalities test (SDMT) z-score^76^.

### Serum neurofilament assessment

Serum neurofilament light chains (sNfL) z-scores were collected within one month of the MRI using a single molecule array assay in 113 patients (86/98 patients with cortical lesions)^59,77^.

### Microstructure estimation

DW-MRI images were denoised^78^ and corrected for motion and eddy-currents^79^. We then applied the novel microstructural diffusion model for SomA and Neurite Density Imaging (SANDI)^39^ to study the cortex of patients and controls.

The SANDI model incorporates water diffusion in spherical objects, which can be assumed to be associated with cell bodies, and in impermeable “sticks” representing neurites. Hence, SANDI allows the characterization of cellular and neurite densities. Details of the model implementation are described in Palombo et al.^39^. Here, we use the Accelerated Microstructure Imaging via Convex Optimization (AMICO)^46^ implementation, named AMICO-SANDI (https://github-wiki-see.page/m/daducci/AMICO/wiki/Fitting-the-SANDI-model), with the following parameters: neurite diffusivity^80^ 2.4 ms/μm^2^, extra-cellular mean diffusivity [0.4, 1.6, 3.0] ms/μm^2^, isotropic-restricted diffusivity 3.0 ms/μm^2^ with nine equally spaced radii in the range 1.5–12 μm. In this study, we focus on the isotropic-restricted compartment.

### Numerical simulation

Numerical simulations were performed using Camino^81^. Substrates were generated with spheres of different radii in the range of 1–15 μm, isotropic-restricted diffusivity 0.1–3.0 ms/μm^2^, signal fraction 10–100%, and SNR = 50. The process was conducted using two different DW-MRI schemes—one from the openly available MGH CONNECTOM DW-MRI scheme used in the original SANDI publication, and the other from the clinical DW-MRI used to acquire the subjects in this study.

Two different experiments were performed: (i) only intra-cellular and (ii) intra-cellular and extra-cellular.

The *only intra-cellular* scenario simulates isotropic-restricted spheres, cellular components, sticks, and neurite components with the above-mentioned parameters. The *intracellular and extra-cellular* scenarios add complexity to the tissue simulation; here, spheres and sticks were fixed to be equal to 1:1, and a third tensor component simulates extra-axonal water.

### Statistical analysis

Statistical analyses were performed using python, R, and FreeSurfer. All p-values are two-tailed.

#### Whole cortex analysis

A Welch’s test was used to assess whether *f*_*soma*_ differed across MS patients’ entire cortex vs. HC.

#### Lesion-wise cortical analysis

Linear mixed-effect models with “patient” as a random effect (since we often have numerous lesions for a single patient) were used to assess whether *f*_*soma*_ was different (i) all patients, (ii) PMS, and (iii) RRMS. Age, sex, medication (untreated/orals/monoclonal antibodies), and lesion type (subpial/leukocortical) were independent covariates.

#### Correlation between *f*_*soma*_ and qMRI

To assess the relationship between *f*_*soma*_ and other quantitative MRI measures, a Pearson correlation was calculated between *f*_*soma*_ and (i) MTsat or (ii) qT1 in CLs.

#### Vertex-wise cortical analysis

A customized volume-to-surface mapping algorithm using Freesurfer V6.0.0 (http://freesurfer.net/fswiki/FreeSurferWiki/) and FSL V6.0 (http://fsl.fmrib.ox.ac.uk/fsl/fslwiki/), was applied to voxels assigned to the grey matter ribbon by FreeSurfer, i.e., voxels with voxel centers located between the white and pial surfaces were registered and scattered into a standard surface. A smoothing kernel of 10-mm full-width at half-maximum was applied. We averaged the *f*_*soma*_ compartment for patients and controls and projected the respective maps to the cortical surface. The inner and outer cortical layers were assessed by sampling up to and from the 50th percentile of the inflated cortex depth. Freesurfer provided a general linear model (GLM) analysis to compare groups with age and sex as covariates. p-values < 0.05 were considered statistically significant, and it has a cluster-wise correction for multiple comparisons (https://surfer.nmr.mgh.harvard.edu/fswiki/FsTutorial/GroupAnalysis).

#### Patient-wise association with clinical scores

We used four linear regression models to assess the relationship between *f*_*soma*_ and (i) EDSS, (ii) MSSS, (iii) SDMT z scores, and (iv) sNFL z-scores. Age (not for the sNFL z-scores), sex (not for the sNFL z-scores), and medication (untreated/oral/monoclonal) were covariates. For sNFL we also added WM volume and diagnosis as covariates.

### Histopathology

The fixed brain of a deceased MS patient (58 years old, secondary progressive MS) was provided by the MS Brain Bank of the German Competence Network Multiple Sclerosis (KKNMS). Tissue blocks were embedded in paraffin, and slices of 4 μm‐thickness were obtained. Immunohistochemical staining was performed using an avidin–biotin technique. Primary antibodies comprised anti-myelin basic protein (anti-MBP; Dako, Glostrup, Denmark for myelin) and anti-CR3/43 (human HLA-DP, clone CR3/43 for MHC-II expressing microglia/macrophages). Double immunofluorescence immunohistochemistry was performed using primary antibodies directed against myelin basic protein (anti-MBP; Dako, Glostrup, Denmark for myelin) and neurofilament proteins cocktail of anti-NF200 (Sigma Aldrich, Missouri, USA), SMI31, SMI32, and SMI311 (Sternberger monoclonals incorporated, Maryland, USA) or astrocytes (cocktail of antibodies against glial fibrillary acidic protein; SYSY, Göttingen, Germany, and Aldh1l1 (aldehyde dehydrogenase 1 family member L1, Merck, Darmstadt, Germany)). Alexa FluorVR488 (Jackson ImmunoResearch Laboratories, Inc.) or CyTM3 (ImmunoResearch Laboratories, Inc.) coupled anti-mouse and anti-rabbit Ig were used as secondary antibodies. DAPI (4ʹ,6-Diamidino-2-phenylindol) was used for nuclear staining.

### Supplementary Information

Supplementary Information.

## Data Availability

The datasets used and/or analysed during the current study available from the corresponding author on reasonable request.
