# Supplementary material for: A novel imaging marker of cortical “cellularity” in multiple sclerosis patients
Source: Sci Rep. 2024 Apr 29;14:9848. doi: 10.1038/s41598-024-60497-6 (PMC11059177; doi:10.1038/s41598-024-60497-6)
Supplement: Supplementary file 1 — Supplementary Information. [file 41598_2024_60497_MOESM1_ESM.docx]

**Supplementary Information**

# **Supplementary Table 1 Surface cortical analysis**

|  | | | **RRMS vs. HC** | | | **PMS vs. HC** | | | **SPMS vs. HC** | | | **RRMS vs. PMS** | | |
| --- | --- | --- | --- | --- | --- | --- | --- | --- | --- | --- | --- | --- | --- | --- |
|  |  |  | Size (mm^2^) | CWP | Annot | Size (mm^2^) | CWP | Annot | Size (mm^2^) | CWP | Annot | Size (mm^2^) | CWP | Annot |
| NEG | left | inner | 26.79  25.83  22.75 | 0.00778  0.01097  0.03391 | Precentral  Postcentral  Parsopercularis | 32.08  27.15 | 0.00100  0.00679 | Lateralorbitofrontal  Superiorfrontal | 22.66 | 0.03509 | Superiorfrontal | 27.62  26.94  26.31  22.99 | 0.00579  0.00719  0.00938  0.03135 | Lateralorbitofrontal  Lateralorbitofrontal  Middletemporal  lateralorbitofrontal |
|  |  | outer | 20.49  20.19 | 0.04130  0.04730 | Superiorparietal  Middletemporal | 22.81 | 0.03391 | Medialorbitofrontal | 49.98  22.45 | 0.00020  0.03803 | Inferiortemporal  Precentral | 36.18  35.07  28.14 | 0.00020  0.00020  0.00479 | Medialorbitofrontal  Lateralorbitofrontal  inferiortemporal |
|  | right | inner | 25.28 | 0.01752 | Superiorparietal | 33.20  24.03 | 0.00060  0.02346 | Insula  Middletemporal | 51.09 | 0.00020 | Postcentral |  |  |  |
|  |  | outer | 24.23 | 0.02366 | Superiorparietal |  |  |  | 28.13  25.30  22.83 | 0.00559  0.01713  0.03960 | Lateraloccipital  Rostralmiddlefrontal  Fusiform | 33.89  23.25 | 0.00100  0.03450 | Lateraloccipital  parahippocampal |
| POS | left | inner | 41.79 | 0.00020 | Temporalpole | 23.07 | 0.03509 | Superiortemporal | 43.80  27.09  22.98 | 0.00020  0.00858  0.03666 | Superiorfrontal  Superiorfrontal  Superiortemporal |  |  |  |
|  |  | outer | 89.34  32.88 | 0.00020  0.00100 | Precentral  Fusiform | 38.74  31.16  27.39  27.00 | 0.00020  0.00140  0.00739  0.00898 | Insula  Precentral  Paracentral  Inferiortemporal | 44.05  37.20  32.24  28.57  26.59  25.04 | 0.00020  0.00020  0.00100  0.00519  0.01097  0.01832 | Insula  Middletemporal  Caudalmiddlefrontal  Precentral  Postcentral  Paracentral | 34.85  25.82  23.34 | 0.00080  0.01216  0.02820 | Insula  Lingual  caudalmiddlefrontal |
|  | right | Inner |  |  |  |  |  |  | 27.21  26.81 | 0.00599  0.00699 | Superiorparietal  Precentral | 27.99 | 0.00499 | insula |
|  |  | outer | 34.83  32.70  31.85 | 0.00040  0.00080  0.00100 | Caudalmiddlefrontal  Inferiorparietal  Lateraloccipital | 54.48  24.49  24.35  23.44  22.64  22.39 | 0.00020  0.01970  0.02109  0.02899  0.04371  0.04879 | Caudalmiddlefrontal  Middletemporal  Insula  Pericalcarine  Superiorfrontal  Insula | 43.10 | 0.00020 | Caudalmiddlefrontal | 26.14 | 0.00958 | insula |

Freesurfer surface cortical analysis

# **Supplementary Table 2 Age-corrected and BMI sNfL z-scores**

| Age (years) | Median (sNfL pg/ml) | median + estimate 5% increase of “cellularity” (sNfL pg/ml) |
| --- | --- | --- |
| 20 | 3.93 | 4.94 |
| 30 | 5.34 | 6.71 |
| 40 | 6.58 | 8.27 |
| 50 | 8.37 | 10.52 |
| 60 | 11 | 13.83 |

In the table, we assumed a BMI of 25. The table shows the relation of sNfL for healthy controls^43^ and patients at different ages. The sNfL for a healthy 20 year old is 3.92 pg/ml. The sNfL for a 20 year old MS patient with a 5% increase in “cellularity” is 4.94 pg/ml. Hence, we can report an addition of 1.02 pg/ml in sNfL.

**Supplementary Table 3 Model parameters**

| **Model EDSS** |  |  |  |  |  |
| --- | --- | --- | --- | --- | --- |
|  | Estimate | Std. Error | t value | Pr(>\|t\|) |  |
| (Intercept) | -0.80602 | 0.78776 | -1.023 | 0.3088 |  |
| Age | 0.08975 | 0.01123 | 7.996 | 3.26E-12 | *** |
| Sexm | -0.18816 | 0.30384 | -0.619 | 0.5372 |  |
| MedicationsOrals | -0.65341 | 0.36534 | -1.789 | 0.0769 | . |
| MedicationsUntreated | -0.30884 | 0.47528 | -0.65 | 0.5174 |  |
| fsoma | 0.84278 | 1.88228 | 0.448 | 0.6554 |  |
|  |  |  |  |  |  |
| Adjusted R-squared: | 0.4145 |  |  |  |  |
| F-statistic: | 15.02 |  |  |  |  |
| p-value: | 8.05E-11 |  |  |  |  |

| **Model MSSS** |  |  |  |  |  |
| --- | --- | --- | --- | --- | --- |
|  | Estimate | Std. Error | t value | Pr(>\|t\|) |  |
| (Intercept) | 3.00057 | 1.16111 | 2.584 | 0.011317 | * |
| Age | 0.06623 | 0.01654 | 4.005 | 0.000125 | *** |
| Sexm | -0.5144 | 0.4494 | -1.145 | 0.255303 |  |
| MedicationsOrals | -1.41976 | 0.53917 | -2.633 | 0.009906 | ** |
| MedicationsUntreated | -1.49105 | 0.70048 | -2.129 | 0.035929 | * |
| fsoma | -2.24492 | 2.77461 | -0.809 | 0.420526 |  |
|  |  |  |  |  |  |
| Adjusted R-squared: | 0.1847 |  |  |  |  |
| F-statistic: | 5.44 |  |  |  |  |
| p-value: | 0.0001956 |  |  |  |  |

| **Model SDMT** |  |  |  |  |  |
| --- | --- | --- | --- | --- | --- |
|  | Estimate | Std. Error | t value | Pr(>\|t\|) |  |
| (Intercept) | 0.466 | 1.258 | 0.370 | 0.714 |  |
| Age | 0.009 | 0.031 | 0.288 | 0.776 |  |
| Sexm | -0.094 | 0.694 | -0.136 | 0.893 |  |
| Wmvolume | -0.00004 | 0.00003 | -1.421 | 0.168 |  |
| MedicationsOrals | -0.296 | 0.751 | -0.394 | 0.697 |  |
| MedicationsUntreated | -0.940 | 1.059 | -0.887 | 0.384 |  |
| fsoma | -2.849 | 2.887 | -0.987 | 0.334 |  |
|  |  |  |  |  |  |
| Adjusted R-squared: | 0.4145 |  |  |  |  |
| F-statistic: | 15.02 |  |  |  |  |
| p-value: | 8.05E-11 |  |  |  |  |

| **NFL zscore (with Age and Sex)** |  |  |  |  |  |
| --- | --- | --- | --- | --- | --- |
|  | Estimate | Std. Error | t value | Pr(>\|t\|) |  |
| (Intercept) | -1.2650696 | 0.9747413 | -1.298 | 0.19939 |  |
| nAge | 0.0003288 | 0.1375539 | 0.002 | 0.9981 |  |
| Sexm | -0.0003216 | 0.2788943 | -0.001 | 0.99908 |  |
| nWMvolume | 0.3338752 | 0.1218986 | 2.739 | 0.00814 | ** |
| DiagnosisRRMS | 0.4691154 | 0.4107722 | 1.142 | 0.25805 |  |
| MedicationsOrals | 0.0882322 | 0.3784383 | 0.233 | 0.81645 |  |
| MedicationsUntreated | 0.2438559 | 0.4099524 | 0.595 | 0.55423 |  |
| f_soma | 0.7375907 | 0.3005875 | 2.454 | 0.01711 | * |
|  |  |  |  |  |  |
| Adjusted R-squared: | 0.1088 |  |  |  |  |
| F-statistic: | 2.612 |  |  |  |  |
| p-value: | 0.03323 |  |  |  |  |
| **NFL zscore (with Age and Sex without f_soma)** |  |  |  |  |  |
|  | Estimate | Std. Error | t value | Pr(>\|t\|) |  |
| (Intercept) | -0.17851 | 0.90397 | -0.197 | 0.8441 |  |
| nAge | 0.00164 | 0.14319 | 0.011 | 0.9909 |  |
| Sexm | 0.04621 | 0.28966 | 0.160 | 0.8738 |  |
| nWMvolume | 0.30563 | 0.12633 | 2.419 | 0.0186 | * |
| DiagnosisRRMS | 0.37206 | 0.42563 | 0.874 | 0.3855 |  |
| MedicationsOrals | -0.04874 | 0.38965 | -0.125 | 0.9009 |  |
| MedicationsUntreated | 0.10035 | 0.42240 | 0.238 | 0.8130 |  |
|  |  |  |  |  |  |
|  |  |  |  |  |  |
| Adjusted R-squared: | 0.001492 |  |  |  |  |
| F-statistic: | 1.016 |  |  |  |  |
| p-value: | 0.4234 |  |  |  |  |

| **NFL zscore (without Age and Sex)** |  |  |  |  |  |
| --- | --- | --- | --- | --- | --- |
|  | Estimate | Std. Error | t value | Pr(>\|t\|) |  |
| (Intercept) | -1.26342 | 0.54783 | -2.306 | 0.02451 | * |
| nWMvolume | 0.33394 | 0.1173 | 2.847 | 0.00601 | ** |
| DiagnosisRRMS | 0.4686 | 0.30814 | 1.521 | 0.13349 |  |
| MedicationsOrals | 0.08837 | 0.36901 | 0.239 | 0.81155 |  |
| MedicationsUntreated | 0.24407 | 0.39116 | 0.624 | 0.53498 |  |
| f_soma | 0.73757 | 0.29493 | 2.501 | 0.01509 | * |
|  |  |  |  |  |  |
| Adjusted R-squared: | 0.1088 |  |  |  |  |
| F-statistic: | 2.612 |  |  |  |  |
| p-value: | 0.03323 |  |  |  |  |
| **NFL zscore (without Age and Sex and f_soma)** |  |  |  |  |  |
|  | Estimate | Std. Error | t value | Pr(>\|t\|) |  |
| (Intercept) | -0.13653 | 0.32448 | -0.421 | 0.6754 |  |
| nWMvolume | 0.30310 | 0.12150 | 2.495 | 0.0153 | * |
| DiagnosisRRMS | 0.35694 | 0.31754 | 1.124 | 0.2653 |  |
| MedicationsOrals | -0.05146 | 0.37989 | -0.135 | 0.8927 |  |
| MedicationsUntreated | 0.10350 | 0.40317 | 0.257 | 0.7983 |  |
|  |  |  |  |  |  |
|  |  |  |  |  |  |
| Adjusted R-squared: | 0.03329 |  |  |  |  |
| F-statistic: | 1.568 |  |  |  |  |
| p-value: | 0.194 |  |  |  |  |

Supplementary Figure 1

***Correlation of soma fraction, extracellular fraction and neurite fraction derived from SANDI with quantitative T1(qT1) and magnetization transfer saturation (MTsat) in subpial (blue) and leukocortical (red) lesions. ‘*’ p<0.05, ‘**’ p<0.01, ‘***’ p<0.001. Error band in the plots are 95% confidence intervals.***


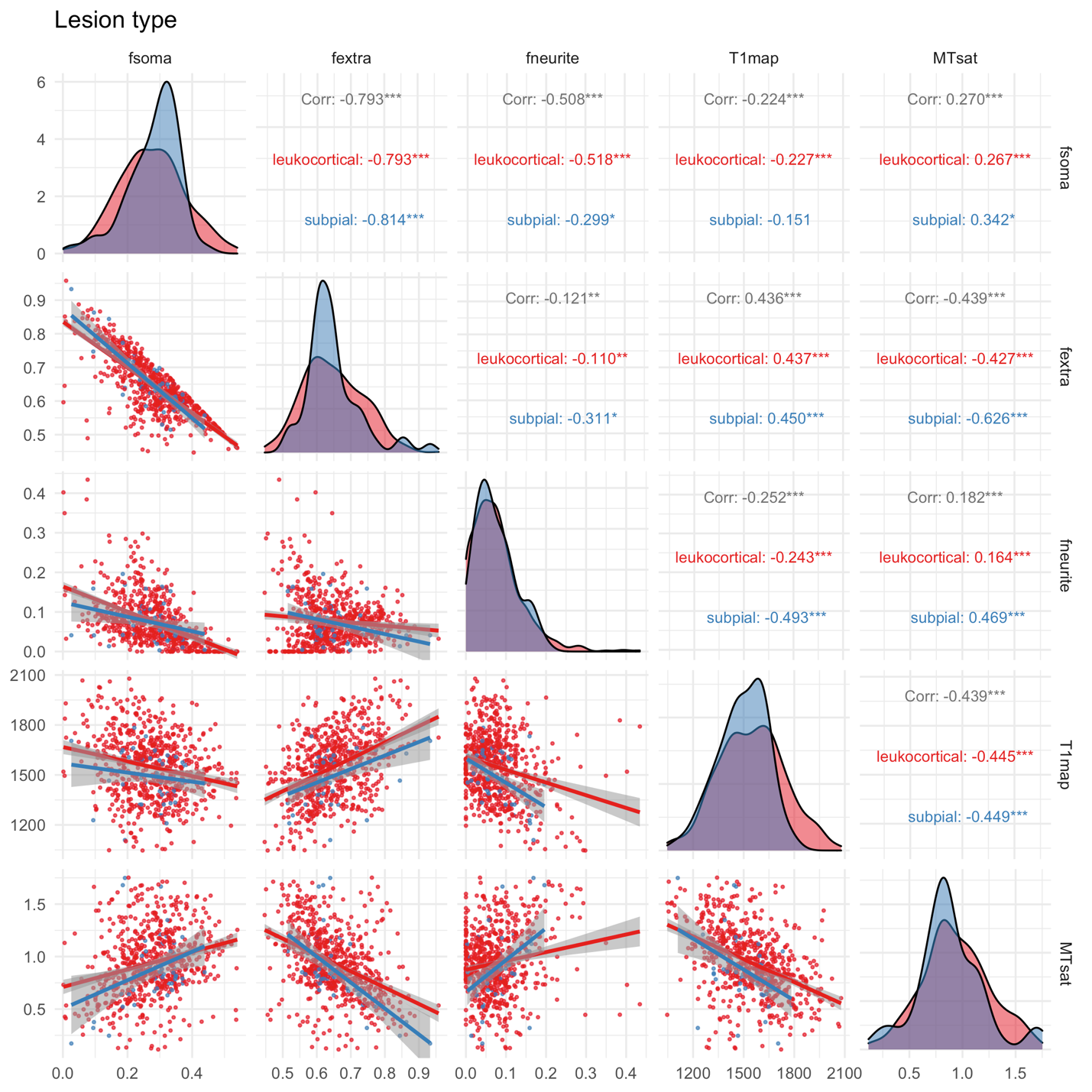


The negative correlation between f_soma_ and f_extracellular_ compartment, as well as between f_soma_ and f_neurite_, as reported in your statement, could be interpreted within the context of the SANDI model as follows:

• A negative correlation between soma and the extracellular compartment could indicate that as the density or volume of the soma increases, there is a corresponding decrease in the relative size or density of the extracellular space. This could be due to the physical space occupied by the soma within the tissue, which reduces the volume available for the extracellular compartment.

• Similarly, a negative correlation between soma and neurite could suggest that an increase in soma density or volume is associated with a decrease in the relative size or density of the neurite compartment. This could be due to the limited physical space within the tissue, where an increase in one cellular component leads to a decrease in another.

The correlations between the extracellular compartment and MTsat and qT1, as well as the correlations between the neurite compartment and these same imaging metrics, can be interpreted in the context of the microstructural properties they represent:

• Extracellular Compartment and MTsat: A negative correlation between the extracellular compartment and MTsat suggests that areas with a larger extracellular space may have less myelin or cellular damage. MTsat is sensitive to the macromolecular content in tissue, including myelin. A larger extracellular space could indicate less dense cellular or myelin content, which would result in lower MTsat values

• Extracellular Compartment and qT1: The positive correlation between the extracellular compartment and qT1 indicates that as the extracellular space increases, the qT1 value also increases. qT1 is a measure of microstructural loss, and a larger extracellular space could be associated with tissue loss or degeneration, which would be reflected in higher qT1 values.

• Neurite Compartment and MTsat: A positive correlation between the neurite compartment and MTsat suggests that areas with a higher density of neurites are associated with higher MTsat values. This is consistent with the understanding that neurites are surrounded by myelin in white matter, and a higher density of neurites would result in a higher content of myelin and other macromolecules, leading to higher MTsat values.

• Neurite Compartment and qT1: The negative correlation between the neurite compartment and qT1 suggests that as the density of neurites increases, the qT1 value decreases. This could be because a higher density of neurites indicates healthier tissue with less microstructural loss, which would be reflected in lower qT1 values.
